# Effects of different harvest frequencies on microbial community and metabolomic properties of annual ryegrass silage

**DOI:** 10.3389/fmicb.2022.971449

**Published:** 2022-08-30

**Authors:** Zhihui Fu, Lin Sun, Meiling Hou, Junfeng Hao, Qiang Lu, Tingyu Liu, Xiuzhen Ren, Yushan Jia, ZhiJun Wang, Gentu Ge

**Affiliations:** ^1^Laboratory of Forage Cultivation, Processing and High Efficient Utilization of Ministry of Agriculture, College of Grassland, Resources and Environment, Hohhot, China; ^2^Key Laboratory of Grassland Resources, Ministry of Education, Inner Mongolia Agricultural University, Hohhot, China; ^3^Inner Mongolia Academy of Agricultural and Animal Husbandry Sciences, Hohhot, China; ^4^College of Life Sciences, Baicheng Normal University, Baicheng, China; ^5^College of Agriculture, Ningxia University, Yinchuan, China; ^6^College of Agriculture, Inner Mongolia Minzu University, Tongliao, China

**Keywords:** ryegrass silage, bacterial community, metabolomics profiles, SMRT, UHPLC-MS/MS

## Abstract

In this study, we analyzed the fermentation quality, microbial community, and metabolome characteristics of ryegrass silage from different harvests (first harvest-AK, second harvest-BK, and third harvest-CK) and analyzed the correlation between fermentative bacteria and metabolites. The bacterial community and metabolomic characteristics were analyzed by single-molecule real-time (SMRT) sequencing and ultra-high-performance liquid chromatography-mass spectrometry (UHPLC-MS/MS), respectively. After 60 days of ensiling, the pH of BK was significantly lower than those of AK and CK, and its lactic acid content was significantly higher than those of AK and CK. *Lactiplantibacillus* and *Enterococcus* genera dominate the microbiota of silage obtained from ryegrass harvested at three different harvests. In addition, the BK group had the highest abundance of *Lactiplantibacillus plantarum* (58.66%), and the CK group had the highest abundance of *Enterococcus faecalis* (42.88%). The most annotated metabolites among the differential metabolites of different harvests were peptides, and eight amino acids were dominant in the composition of the identified peptides. In the ryegrass silage, arginine, alanine, aspartate, and glutamate biosynthesis had the highest enrichment ratio in the metabolic pathway of KEGG pathway enrichment analysis. Valyl-isoleucine and glutamylvaline were positively correlated with *Lactiplantibacillus plantarum*. D-Pipecolic acid and L-glutamic acid were positively correlated with *Levilactobacillus brevis*. L-phenylalanyl-L-proline, 3,4,5-trihydroxy-6-(2-methoxybenzoyloxy) oxane-2-carboxylic acid, and shikimic acid were negatively correlated with *Levilactobacillus brevis*. In conclusion, this study explains the effects of different harvest frequencies on the fermentation quality, microbial community, and metabolites of ryegrass, and improves our understanding of the ensiling mechanisms associated with different ryegrass harvesting frequencies.

## Introduction

Annual ryegrass (*Lolium multiflorum* Lam.) is a globally significant forage crop. Because of its high nutritional value, digestibility, and well ensiling characteristics, it is now widely distributed throughout the temperate areas of Europe, America, and Asia. It is commonly regarded as the popular feed source for herbivorous animals during the winter season (Parvin et al., 2010; Dong et al., 2020; Lv et al., 2021). Annual ryegrass is one of the most important forages for dairy cows in temperate regions (Smit et al., 2005), and is also widely sown for grazing ruminants due to its high forage yield and nutritive value, particularly high soluble and degradable N and carbohydrates (Stergiadis et al., 2015). Ensiling is a traditional and effective method of preserving feed for long periods of time. Lactic acid bacteria (LAB) proliferate and produce organic acids in an anaerobic environment, significantly lowering the pH of the feed and suppressing the activities of dangerous bacteria (Ren et al., 2020). It also helps to bridge the seasonal imbalance between livestock feed demand and available high-quality fodder by extending the stable storage duration of forage (Wright et al., 2000). Ensiling is a fermentation process involving microbial community succession in which multiple types of fermenting microorganisms produce a variety of metabolites that affect forage utilization and storage, as well as animal production performance (Kung et al., 2018; Wang et al., 2022a). Several studies have used integrative 16S rRNA sequencing and metabolomics to analyze silage microbiome and metabolome conditions to better understand the biological mechanisms underpinning silage production (Guo et al., 2018; Xu et al., 2019). However, shorter genomes with relatively low taxonomical resolution have limited the classification of microorganisms in the community to the genus level (Amir et al., 2013). Long reads can be generated by Pacific Biosciences’ (PacBio) third-generation, single-molecule, real-time (SMRT) sequencing technology, which improves classification sensitivity and accuracy while allowing for relatively high taxonomic resolution down to the species level (Hou et al., 2015; Schloss et al., 2016). Metabolomics research tools help to reveal the biochemical network mechanisms of the fermentation process, and metabolomics techniques can be used to guide the regulation and prediction of component changes in the fermentation process. A large number of metabolites, including various amino acids, fatty acids, oligosaccharides, vitamins, peptides, and aromatic substances, are produced during the fermentation process. The study of the types, quantities, and influencing factors of these metabolites is important for the scientific evaluation and utilization in silage fermentation involving LAB. The identification of differential and specific biomarkers from a large number of metabolites has become a research hotspot in metabolomics (Sun et al., 2012). Therefore, the integrated analysis of the metabolome and microbiome allows for better identification of unknown metabolites, new functional LAB, and the corresponding metabolic processes during ensiling.

To our knowledge, few studies have analyzed the effects of different harvest frequencies on the quality of ryegrass silage fermentation and fermentation mechanisms using multiomics techniques, and no previous work used LC-MS to identify the metabolome of ryegrass silage. We hypothesized that there would be different ensiling mechanisms for different harvests when ryegrass was harvested more than once during a growing season. Therefore, the current study aimed to investigate the effect of different harvests on silage quality by SMRT sequencing and metabolomics, and to reveal the effect of different harvesting frequencies on the fermentation mechanism of ryegrass silage from the perspective of microbiology and metabolomics. In addition, understanding the relationship between metabolites and fermenting bacteria can provide a deeper understanding of the fermentation mechanism of silage, thus providing new ideas for the screening and utilization of LAB and a theoretical basis for improving the quality of fermentation.

## Materials and methods

### Experimental design and mini silos preparation

The study was carried out from May to August 2021 (May–August: monthly mean temperatures: 15.3°C, 21.0°C, 23.2°C, and 19.3°C; monthly total precipitation: 7.1, 55.6, 122.6, and 111.7 mm) at the forage experiment site of Inner Mongolia Agricultural University (111°43′E, 40°48′N, altitude 1,056 m above sea level, Hohhot, Inner Mongolia). Six plots (15 × 7 m^2^; each plot separated by 0.5 m) of ryegrass were planted on May 12 on chestnut caliche soil. Ryegrass was harvested three times in total. The first (A), second (B), and third (C) harvests were on July 7, July 27, and August 16, respectively. Three plots of fresh ryegrass were randomly chosen and selected for sample collection at the ryegrass booting period, and the collected samples were placed on clean plastic sheets and left to sun-dry. As they dried, we measured the moisture until dry matter content at about 35% was achieved, whereupon the ryegrass was cut into about 3 cm sections with a guillotine (Type: Mode-8,200; Minghong Business, Shandong, China). The chopped ryegrass from each plot was fully mixed and divided into three equal portions and used as replicates. Each portion (300 g) was collected in sterilized bags and placed in an icebox, and then immediately transported to the laboratory at the same time. We evenly divided each (300 g) sample into two equal parts (3 replicates*2 = 6 repeats), put 20 g of each sample into a liquid nitrogen tank, and immediately sent them to the laboratory for storage in a −80°C refrigerator. These samples were sent to Majorbio Bio-Pharm Technology (Majorbio Bio-Pharm Technology Co., Ltd., Shanghai, China) to perform the metabolome analysis. The chemical composition and microbial population of fresh ryegrass were evaluated (Table 1). We then packed the rest of the prepared ryegrass (500 g) in polyethylene plastic bags (size: 300 mm × 400 mm; smooth food vacuum bag; Shenyang Huasheng Plastic Packaging Products Co., Ltd., China) and vacuum sealed them with a vacuum sealer (Type: DZ-500/2E; Hefei Hanjie Packaging Machinery Inkjet Co., Ltd., Hefei, China). In addition, no additives were added to these samples. All bags (3 treatments × 3 repeats) were then stored at ambient temperature (24–26°C) under sheltered conditions. Silage quality, microbial community, and metabolites were measured for the first (AK), second (BK), and third (CK) ryegrass harvests after 60 days of ensiling.

**TABLE 1:** Chemical and microbial compositions of fresh ryegrass.

Items	Harvest			SEM	*P*-value
			
	First	Second	Third		
DM, g/kg	354.02	350.46	341.27	15.85	0.713
CP, g/kg DM	172.50a	145.23b	141.31b	1.52	0.002
NDF, g/kg DM	503.60b	532.40a	530.57a	1.05	0.018
ADF, g/kg DM	304.70b	330.13a	324.30a	12.04	0.007
WSC, g/kg DM	86.30a	66.53c	77.40b	8.86	0.004
Lactic acid bacteria (log_10_ cfu/g FM)	4.73	6.09	4.21	1.31	0.627
Coliform bacteria (log_10_ cfu/g FM)	5.89	7.19	5.69	0.92	0.221
Yeast (log_10_ cfu/g FM)	3.44b	6.85a	6.87a	0.09	<0.001
Molds (log_10_ cfu/g FM)	4.05b	4.71a	4.73a	0.08	0.079

FM, fresh matter; DM, dry matter; CP, crude protein; NDF, neutral detergent fiber; ADF, acid detergent fiber; WSC, water-soluble carbohydrate; SEM, standard error of the mean; cfu, colony-forming unit; Mean values with different letters in the same row (a–c) differ significantly (P < 0.05).

### Sample collection and measurements

Three parallel determinations were performed for each sample of fresh and silage ryegrass, including chemical composition, fermentation characteristics, and microbial counts. Following the method of Zhang et al. (2016), the dry matter (DM) content was determined by oven drying at 65°C for 48 h. The crude protein content was determined using a Kjeldahl apparatus (Gerhart Vapodest 50 s, Germany) following Patrica (1997). The neutral detergent fiber (NDF) and acid detergent fiber (ADF) were measured according to Van Soest procedures (Van et al., 1991), using an Ankom A2000i fiber analyzer (A2000i, Ankom Technology, Macedon, NY, United States). The water-soluble carbohydrate (WSC) content was determined according to the methods of Chen et al. (2015).

The fermentation products of silage were analyzed using cold water extract. A sample (10 g) of silage was combined with 90 g of deionized water and stored in a 4°C refrigerator for 24 h as described by Cai (2004). Four layers of cheesecloth and filtered paper were used to filter the liquid extract. The pH, ammonia nitrogen (NH3-N), and organic acids were measured in the produced filtrates. A glass electrode pH meter was used to measure the pH (LEICI pH S-3C, Shanghai, China). Following Cheng et al. (2021), the lactic acid (LA), acetic acid (AA), propionic acid (PA), and butyric acid (BA) content of silage was determined using high-performance liquid chromatography. Broderick and Kang’s (1980) approach was used to determine the nitrogen (NH3-N) concentration.

Referring to Sun et al. (2021), 10 g of fresh or silage ryegrass was blended with 90 ml of sterilized water, and the extract was serially diluted to quantify the bacterial group in a sterile solution, and the numbers of LAB, coliform bacteria, yeast, and molds were measured. Culture media (Guangzhou Huankai Microbial Science and Technology Co., Ltd., Guangzhou, China) were used to isolate and enumerate various microorganisms. The plates for enumerating LAB (De Man Rogosa Sharpe agar culture media) were placed in an anaerobic box (Anaerobic box; C-31, Mitsubishi Gas Chemical Co., Tokyo, Japan) and incubated at 37°C for 48 h. The plates for coliform bacteria (violet-red bile agar culture media) were incubated at 37°C for 48 h, and yeast and molds (potato dextrose agar culture media) were incubated at 30°C for 48 h in aerobic conditions in a general incubator (GP-01, Huangshi Hengfeng Medical Instrument Co., Ltd., Huangshi, China).

### Sequencing and analysis of microbial diversity

The genomic DNA of microbial community inhabiting fresh and silage ryegrass samples was extracted using the FastDNA SPIN for soil kit (MP Biomedicals, Solon, United States), and a NanoDrop 2000 UV-Vis spectrophotometer (Thermo Fisher Scientific, Wilmington, United States) was used to assess the concentration and purity of the DNA. The primer pairs 27F and 1,492 R, which span the full length of the bacterial 16 S rRNA gene, were amplified using an ABI GeneAmp 9700 PCR thermocycler (ABI, CA, United States). The PCR amplification was performed following the methods of Li et al. (2022).

The PCR result was extracted from 2% agarose gel, and the PCR products were purified using the AMPure^®^ PB beads (Pacifc Biosciences, CA, United States) and quantified with Quantus Fluorometer (Promega, WI, United States). Purified products were pooled in equimolar amounts, and a DNA library was constructed using the SMRTbell^®^ Express Template Prep Kit 2.0 (Pacifc Biosciences, CA, United States) according to the manufacturer’s instructions. Purified SMRTbell libraries were sequenced on the Pacbio Sequel II System (Pacifc Biosciences, CA, United States) and using single-molecule real-time (SMRT) sequencing technology (Majorbio Bio-Pharm Technology Co., Ltd., Shanghai, China). FLASH was used to assemble the raw 16S rRNA gene sequencing data (Magoc and Salzberg, 2011). UPARSE was used to cluster operational taxonomic units (OTUs) using a 97% similarity criterion. RDP classifier (Version 2.2) (Wang et al., 2007) was used to assess the taxonomy of each OTU representative sequence against the database (nt_v20210917), using a confidence threshold of 0.7.

### Sequencing and analysis of metabolites

The metabolites were extracted from a 50 mg solid ensiled ryegrass sample in EP tubes using a 400 L methanol: water (4:1, v/v) solution, following the methods of Zhang et al. (2019). As part of the system conditioning and quality control process, a pooled quality control sample (QC) was prepared by mixing equal volumes of all samples. The QC samples were treated and tested in the same manner as the analytic samples (3 treatments × 6 repeats = 27). The QC samples were injected at regular intervals (every six samples) in order to monitor the stability of the analysis. Ultra-high-performance liquid chromatography-tandem Fourier transform mass spectrometry UHPLC-Q Exactive HF-X system from Thermo Fisher served as the apparatus platform for this LC-MS study. Ultra-high-performance liquid chromatography-mass spectrometry (UHPLC-MS/MS) analyses were performed following Yang et al. (2022).

### Multivariate statistical analysis of metabolites and identification of differential metabolites

The ropls (Version 1.6.2) R package was used to perform multivariate statistical analysis. To gain an overview of the metabolic data, an unsupervised approach of principle component analysis (PCA) was used to depict general clustering, trends, and outliers. Prior to the PCA, all of the metabolite variables were scaled to unit variances. A total of 375 differential metabolites in three groups were summarized using one-way analysis of variance (ANOVA) for multiple comparisons of silage samples from the three harvests by screening for differential metabolites with asymptotic statistical significance (*p* < 0.05) to create metabolic sets. Metabolic enrichment and pathway analyses were built up and connected to biochemical pathways using database searches (KEGG).^1^ These metabolites can be classified into categories based on the pathways they took or the functions they played. Enrichment analysis is frequently used to identify whether or not a group of metabolites in a function node appeared. The notion is that an investigation of a single metabolite would lead to an analysis of a group of metabolites. The scipy.stats (Python packages)^2^ was used to find statistically significantly enriched pathways using Fisher’s exact test.

### Statistical analysis

The fermentation, nutritional characteristics, and microbial counts of fresh and silage ryegrass were analyzed using a one-way analysis of variance (ANOVA) based on the general linear model (GLM) procedure of SAS (version 9.3; SAS Institute Inc., Cary, NC, United States). One-way analysis of variance (ANOVA) and Duncan’s multiple range test were used to evaluate differences among treatments, and the effect was considered significant when *p* < 0.05. Microbiota and metabolome data were performed using an online platform of Majorbio I-Sanger Cloud Platform.^3^

## Results

### Chemical and microbial compositions of fresh ryegrass materials

The chemical composition and microbial populations of fresh ryegrass before ensiling are shown in Table 1. The DM content of fresh ryegrass ranged from 341.27to 354.02 g/kg. The CP content (172.50 g/kg) of the first harvest sample was significantly higher than at other harvests (*p* < 0.05). The NDF and ADF content of the first harvest sample was significantly lower than at other harvests, and its WSC was significantly higher (*p* < 0.05). There was no significant difference in LAB or coliform bacteria among harvests, but the yeast and mold content of the first harvest sample was significantly lower than at later harvests (*p* < 0.05).

### Silage quality of different harvest ryegrass silage

Silage quality after 60 days of different harvests of ryegrass is presented in Table 2. After 60 days of ensiling, there were significant effects of harvest on silage AN/TN, acetic acid, and propionic acid (*p* < 0.05). The pH of the silages ranged from 4.43 to 5.44, and the second harvest silage achieved a significantly lower pH value (4.43) compared to the others (*p* < 0.05). The WSC content (65.63 g/kg) of the third harvest silage was significantly higher than the others (*p* < 0.05). In addition, across the three harvests, the pH and acetic acid concentration first decreased and then increased, but the lactic acid, propionic acid, and butyric acid concentrations showed the opposite trend.

**TABLE 2:** Silage quality of ryegrass silage in different harvests.

Items	Harvest			SEM	*P*-value
			
	First	Second	Third		
pH	4.85b	4.43c	5.44a	0.05	<0.001
AN/TN,%	3.13	5.50	1.87	2.08	0.243
Lactic acid, g/kg DM	84.98b	130.68a	54.01c	33.81	0.006
Acetic acid, g/kg DM	11.74	11.06	12.44	3.86	0.899
Propionic acid, g/kg DM	19.56	29.23	18.60	6.71	0.213
Butyric acid, g/kg DM	10.04*ab*	12.23a	6.36b	3.17	0.156
DM, g/kg FM	335.10a	322.43b	336.73a	4.44	0.143
WSC, g/kg DM	55.63b	43.27c	65.63a	2.73	0.002

FM, fresh matter; DM, dry matter; AN/TN, ammonia nitrogen/total nitrogen; WSC, water-soluble carbohydrate; SEM, standard error of the mean; Mean values with different letters in the same row (a–c) differ significantly (P < 0.05).

### Bacterial community and metabolomic profiles of ryegrass silage

As shown in Table 3, based on SMRT sequencing of the full-length 16S rRNA genes of fresh materials and ryegrass silage bacteria, all the samples had coverage values better than 99%, indicating that the sequencing depth was adequate for valid bacterial community characterization. After ensiling, the Sobs, Shannon, Ace, and Chao 1 values ranged from 39.00 to 59.00, 1.45 to 2.53, 68.62 to 98.50, and 51.06 to 82.00, respectively. It follows that the harvest number greatly affected the bacterial community of the resultant silage. Compared with fresh forage, the Sobs, Shannon, Ace, and Chao 1 values in each treated silage decreased. In addition, the highest Sobs, Shannon, and Ace values of bacterial diversity and community were observed in the third harvest of fresh materials.

**TABLE 3:** Alpha-diversity of the bacterial community in fresh materials and ryegrass silage.

Treatment	Sobs	Shannon	Ace	Chao 1	Coverage
First					
0 day	140.67	3.88	153.34	155.00	0.996
60 day	59.00	2.53	86.36	80.25	0.997
Second					
0 day	129.00	3.40	139.01	136.95	0.996
60 day	39.00	1.45	68.62	51.06	0.998
Third					
0 day	141.33	4.05	153.79	154.11	0.996
60 day	57.00	2.34	98.50	82.00	0.996

**FIGURE 1:**
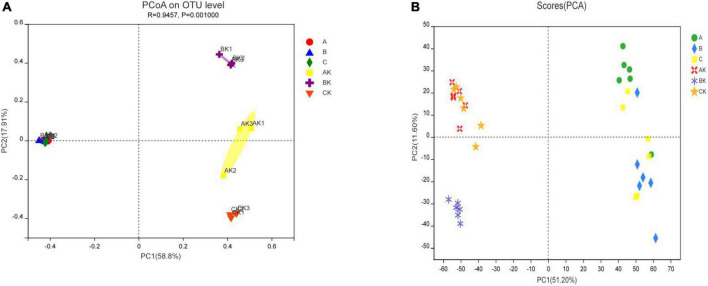
The principal coordinate analysis (PCoA) plot showing variation in bacterial community structure of different harvests. **(A)** The principal component analysis (PCA) of metabolites showing variation among the different harvests of fresh and silage ryegrass. **(B)** Each point represents an individual. A, first harvest of fresh ryegrass; B, second harvest of fresh ryegrass; C, third harvest of fresh ryegrass; AK, First harvest of ryegrass silage; BK, second harvest of ryegrass silage; CK, third harvest of ryegrass silage.

A β-diversity analysis utilizing principal coordinates analysis (PCoA) was used to confirm the differences in the bacterial communities of the fresh materials and ryegrass silage (Figure 1A). The PCoA was based on Bray-Curtis distances at the OTU level and an ANOSIM test with 999 permutations between different groups. As shown in Figure 1A, we observed that there was no significant separation between the bacterial communities of fresh ryegrass materials, but after ensiling, the bacterial communities of different harvests had clearly separated epiphytic microbial communities. For the metabolomic features, no significant separation was found in fresh ryegrass materials (A, B, C) according to the PCA plot (Figure 1B). However, separation was found between AK, CK, and BK silage treatments, although a good separation between AK and CK was not observed.

### Bacterial community changes in ryegrass silage

**FIGURE 2:**
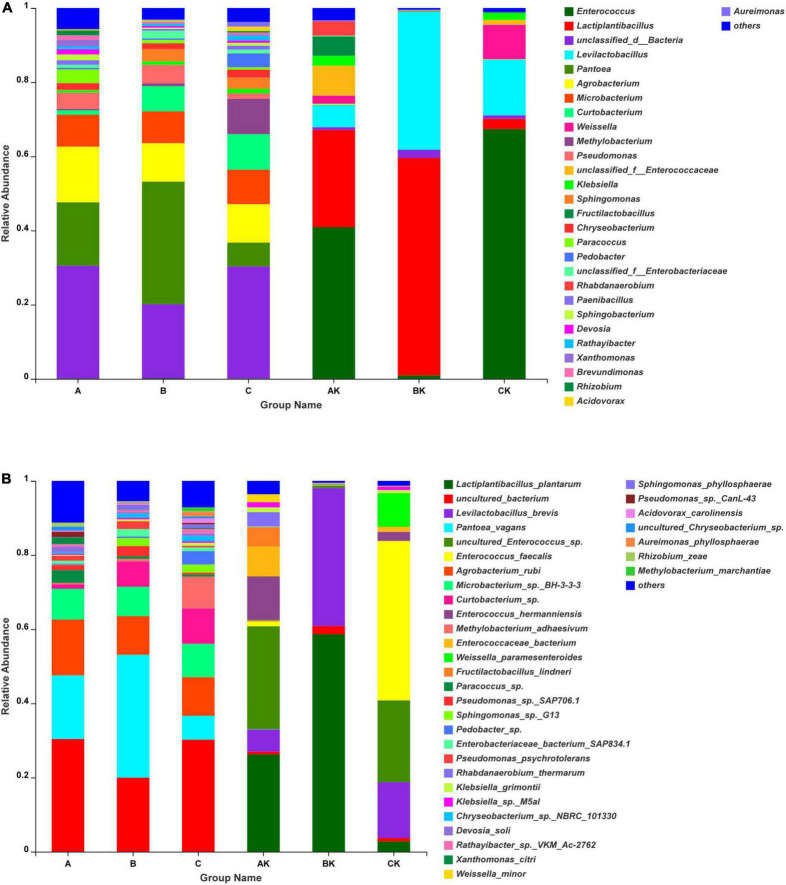
Bacterial community composition at genus **(A)** and species **(B)** levels in fresh ryegrass and ryegrass silage by SMRT. A, first harvest of fresh ryegrass; B, second harvest of fresh ryegrass; C, third harvest of fresh ryegrass; AK, first harvest of ryegrass silage; BK, second harvest of ryegrass silage; CK, third harvest of ryegrass silage.

The dynamics of bacterial communities in different harvests of fresh ryegrass and ryegrass silage at the genus level revealed by SMRT is described in Figure 2A. *Pantoea* and unclassified bacteria were the main genera in the fresh materials. *Enterococcus* and *Lactiplantibacillus* were the most common bacteria in the AK, CK, and BK groups during the ensiling process. However, the community composition was influenced by harvest number. The highest abundance of *Lactiplantibacillus* (58.66%) was in BK, and the highest abundance of *Enterococcus* (40.88 and 67.36%) was in AK and CK. The relative abundance of fresh ryegrass and ryegrass silage bacteria at the species level by SMRT is shown in Figure 2B. Uncultured bacteria (A: 30.41%; C: 30.23%) and *Pantoea vagans* (C: 33.08%) were the main species in fresh ryegrass. *Lactiplantibacillus plantarum* was undetectable in fresh materials. After 60 days of the ensiling process, the uncultured *Enterococcus* sp., *Lactiplantibacillus plantarum*, and *Enterococcus faecalis* were the most dominant species in the AK, BK, and CK groups, respectively. Their abundances were 27.76, 58.66, and 42.88%, respectively.

**FIGURE 3:**
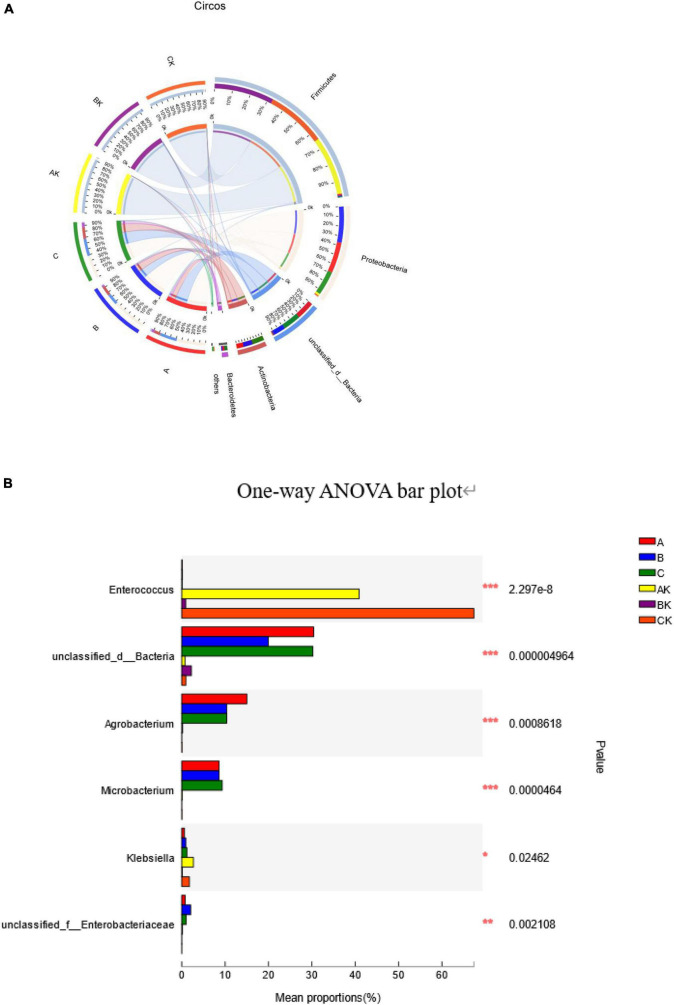
Differences in the relative abundance of the microbial community at the phylum level shown by a Circos plot. The size of the bars from each phylum shows the relative abundance of that phylum in sample **(A)**. One-way analysis of variance bar plots at the genus level (10 most abundant genera) among the fresh and silage ryegrass. *p < 0.05; **p < 0.01; ***p < 0.001 **(B).** A, first harvest of fresh ryegrass; B, second harvest of fresh ryegrass; C, third harvest of fresh ryegrass; AK, first harvest of ryegrass silage; BK, second harvest of ryegrass silage; CK, third harvest of ryegrass silage.

The succession of bacterial community dynamics at the phylum level during the ensiling of ryegrass is shown in Figure 3. The dominant phyla in fresh ryegrass microbiota were Proteobacteria (A: 50.12%; B: 59.38%; and C: 41.70%) and unclassified bacteria (A: 30.43%; B: 19.89%; and C: 31.63%). However, during the ensiling of ryegrass, the AK microbiota was dominated by Firmicutes (95.20%) and Proteobacteria (3.83%); the BK microbiota was dominated by Firmicutes (98.08%) and unclassified bacteria (1.63%); and the CK microbiota was dominated by Firmicutes (97.02%) and Proteobacteria (1.70%) (Figure 3A). Figure 3B illustrates the dynamics of bacterial populations in fresh and silage ryegrass at the genus level. The main epiphytic genus of group A was unclassified bacteria and *Agrobacterium*, and the main epiphytic genus of group C was *Microbacterium*, which was very significantly higher than in the other groups (*p* < 0.001). Their abundance decreased greatly during the ensiling process. After ensiling ryegrass, significantly different dominant genera were *Enterococcus*, unclassified bacteria, and *Klebsiella.* In addition, the main genus was *Enterococcus* in the AK (40.88%) and CK (67.36%) groups, and their abundance was significantly higher than that of other genera (*p* < 0.001).

**FIGURE 4:**
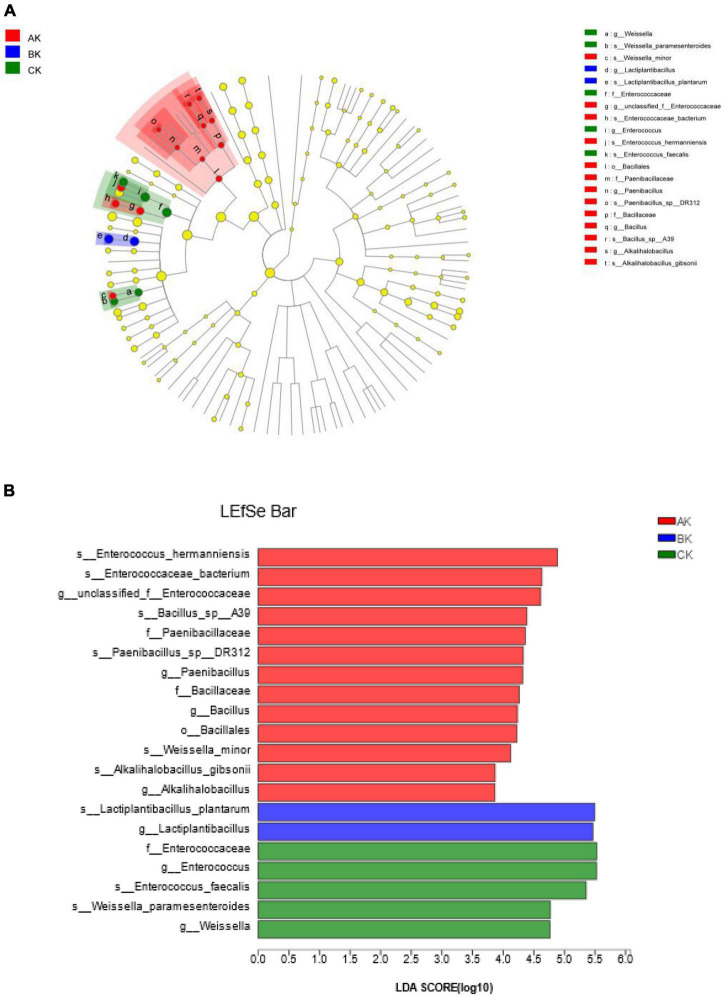
Cladogram showing the phylogenetic distribution of bacteria after 60 days of silage at different harvests. **(A)** Indicator bacteria with an LDA score of 2 or more in the silage bacterial community under different treatments. **(B)** Areas of different colors represent different treatments (red, AK; blue, BK; green, CK). Circles indicate the phylogenetic level from the kingdom to the species level. Each node (small circle) represents a taxonomic unit. Yellow nodes indicate that the two groups of bacteria were not statistically or biologically different. The diameter of each circle is proportional to the abundance of the group.

We used LEfSe to identify specific communities in the sample, and only statistical analyses from the kingdom to species level were performed in this study. Cladograms depicted several treatments, and LEfSe verified LDA values of 2 or greater (Figure 4). In AK, 13 groups of bacteria were significantly enriched, and *Enterococcus hermanniensis* (4.89) had the largest LDA scores. In BK, two groups of bacteria were significantly enriched, and *Lactiplantibacillus plantarum* (5.50) had the largest LDA scores. In CK, five groups of bacteria were significantly enriched, and *Enterococcaceae* (5.53) had the largest LDA scores. These results indicated that species abundance differs in specific communities at different harvests.

### Identification and analysis of metabolites

**FIGURE 5:**
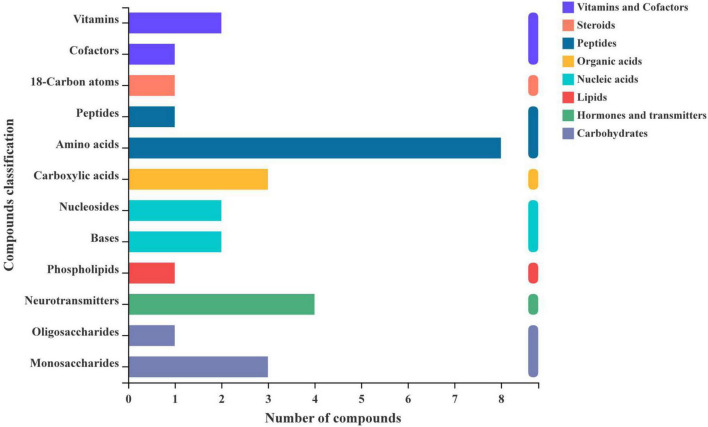
Comparison of the classification of KEGG compounds at different harvests.

To further understand the compounds produced by silage metabolism, we used KEGG compound classification to analyze the classification of metabolites under different harvests (Figure 5). According to their biological roles, metabolites were divided into lipids, carbohydrates, nucleic acids, peptides, vitamins and cofactors, steroids, hormones and transmitters, and organic acids. After 60 days of ensiling, the most annotated metabolites were peptides, and eight amino acids were dominant in the composition of the identified peptides. In addition, two nucleosides and two bases were annotated, which were nucleic acids. Four neurotransmitters were annotated and they were hormones and transmitters. One oligosaccharide and monosaccharide were annotated, which were carbohydrates.

**FIGURE 6:**
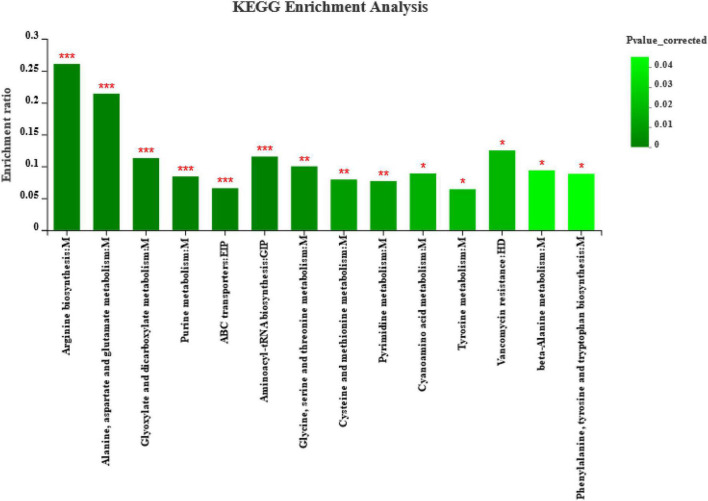
Metabolite pathway enrichment analysis following positive and negative mode ionization. Overview of metabolites enriched in ryegrass after silage of different harvests. M, EIP, GIP, and HD are the class names of metabolic pathways in KEGG annotation. M, Metabolism; EIP, Environmental Information Processing; GIP, Genetic Information Processing; HD, Human Diseases. *p*-value-corrected < 0.05 and column chart showing *p*-values for the top 20 pathways; *p < 0.05; **p < 0.01; ***p < 0.001.

Furthermore, enrichment analysis (Figure 6) showed that metabolic pathways, such as arginine biosynthesis, alanine, aspartate, and glutamate metabolism, glyoxylate and dicarboxylate metabolism, purine metabolism, ABC transporters, and aminoacyl-tRNA biosynthesis, were significantly affected (*p* < 0.001) by different harvests. The highest enrichment rates were for arginine biosynthesis, alanine, aspartate, and glutamate metabolism. The ABC transporters are a pathway related to environmental information processing, and the alanine, aspartate, and glutamate metabolism are a pathway related to genetic information processing according to the KEGG Pathway database classification. In addition, metabolic pathways of fermenting bacteria, such as glycine, serine, and threonine metabolism, cysteine and methionine metabolism, and pyrimidine metabolism, were significantly affected (*p* < 0.01) by different harvests. Metabolic pathways, such as cyanoamino acid metabolism, tyrosine metabolism, vancomycin resistance, beta-alanine metabolism, and phenylalanine, tyrosine, and tryptophan biosynthesis, were significantly affected (*p* < 0.05) by different harvests, among which the vancomycin resistance is a pathway related to human diseases according to KEGG Pathway database classification.

### Correlations between the relative abundance of bacteria and metabolites

**FIGURE 7:**
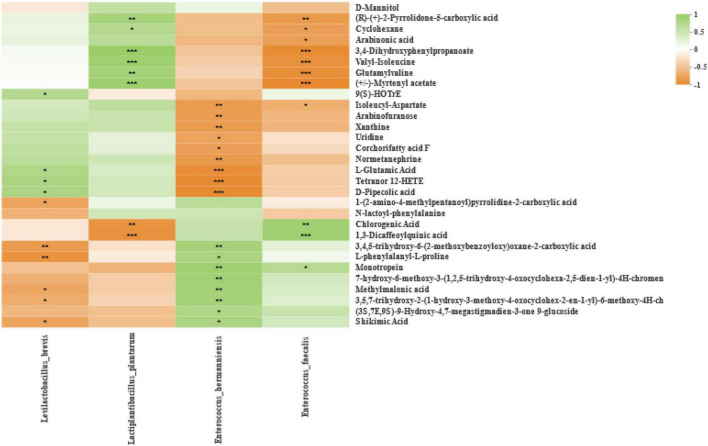
Correlation analysis of the high abundance of species-level bacteria and metabolites in silage from different harvests. *p < 0.05, **p < 0.01, and ***p < 0.001, respectively.

Spearman correlation between bacterial and differential metabolites at the level of species is shown in Figure 7, and bacteria in silage of different harvests that were co-occurring or highly enriched were classified and named. Correlation analysis indicated negative and positive correlations (*p*-values are shown as *p < 0.05, ^**^p < 0.01, ^***^p < 0.001). After 60 days of ensiling, (R)-(+)-2-pyrrolidone-5-carboxylic acid and cyclohexane were positively correlated with *Lactiplantibacillus plantarum*, and negatively correlated with *Enterococcus faecalis*. Arabinonic acid was negatively correlated with *Enterococcus faecalis*. Glutamylvaline, 3,4-dihydroxyphenylpropanoate, valyl-isoleucine, and myrtenyl acetate were positively correlated with *Lactiplantibacillus plantarum*, and negatively correlated with *Enterococcus faecalis*. 9(S)-HOTrE was positively correlated with *Levilactobacillus brevis*. Isoleucyl aspartate was negatively correlated with *Enterococcus hermanniensis* and *Enterococcus faecalis*. Arabinofuranose, xanthine, uridine, corchorifatty acid F, and normetanephrine were negatively correlated with *Enterococcus hermanniensis*. The L-glutamic acid, tetranor 12-HETE, and D-pipecolic acid were positively correlated with *Levilactobacillus brevis*, and negatively correlated with *Enterococcus hermanniensis*. 1-(2-Amino-4-methylpentanoyl)pyrrolidine-2-carboxylic acid was negatively correlated with *Levilactobacillus brevis*. Chlorogenic acid and 1,3-dicaffeoylquinic acid were negatively correlated with *Lactiplantibacillus plantarum*, and positively correlated with *Enterococcus faecalis*. 3,4,5-Trihydroxy-6-(2-methoxybenzoyloxy)oxane-2-carboxylic acid and L-phenylalanyl-L-proline were negatively correlated with *Levilactobacillus brevis*, and positively correlated with *Enterococcus hermanniensis*. Monotropein was positively correlated with *Enterococcus hermanniensis* and *Enterococcus faecalis*. 7-Hydroxy-6-methoxy-3-(1,2,5-trihydroxy-4-oxocyclohexa-2,5-dien-1-yl)-4H-chromen and (3S,7E,9S)-9-hydroxy-4,7-megastigmadien-3-one 9-glucoside were positively correlated with *Enterococcus hermanniensis*. Methylmalonic acid, 3,5,7-trihydroxy-2-(1-hydroxy-3-methoxy-4-oxocyclohex-2-en-1-yl)-6-methoxy-4H-ch, and shikimic acid were negatively correlated with *Levilactobacillus brevis*, and positively correlated with *Enterococcus hermanniensis*.

## Discussion

The amount of epiphytic LAB in raw materials is critical for the success of silage (Cai et al., 1999). According to Wang et al. (2017), the minimum number of LAB in the raw material should be greater than 5.0 log_10_ CFU per gram FM. The second harvest of fresh ryegrass had a higher number(6.09 log_10_ CFU per gram FM)of LAB to meet the fermentation requirements, while the first (4.73 log_10_ CFU per gram FM) and third (4.21 log_10_ CFU per gram FM) harvests had slightly lower numbers and higher concentrations of unfavorable microorganisms (> 6.41 log_10_ CFU per gram FM), including yeasts, aerobic bacteria, and coliform bacteria, which might contribute to unacceptable silage fermentation. Moreover, non-structural carbohydrates (NSC), which comprise soluble carbohydrates (e.g., sucrose and fructose) and starch, are the most essential sources of energy in the plant development and metabolic process (Wang et al., 2017). The amount of WSC in the raw material is important for lactic acid fermentation, and the ideal WSC concentration for silage fermentation is at least 50 g/kg DM (Li et al., 2019). The WSC content (86.30 g/kg, 66.53 g/kg, and 77.40 g/kg) of different harvests of fresh ryegrass met that threshold, showing that microorganisms had enough substrate.

Ensiling is a complicated bacterial fermentation process that results in the accumulation of organic acid and a decrease in pH (Ding et al., 2020). The acidity of silage is crucial for stability, and a pH below 5.0 favors the proliferation of desirable LAB and limits the growth of undesirable microorganisms (Keles et al., 2014). A pH of 4.2 or below indicates that the silage is well-fermented (Mcdonald et al., 1991). The pH of ryegrass without the addition of exogenous ferment was generally higher than 4.2 after natural fermentation (Table 2), as was also found by Wang S. et al. (2018). After ensiling, all the silages had consistently lower DM levels than the fresh material in the current study. The results of this study were in agreement with the reports that the DM content after ensiling was lower than that of the raw material before ensiling in all cases. This may be due to the fact that during the fermentation process, the easily degradable carbohydrates (WSC) of silage were transformed into silage acids, ethanol, and carbon dioxide by fermenting microorganisms (Wang S. et al., 2018). It is possible that the metabolism of soluble substances (e.g., WSC) by fermenting microorganisms may lead to a reduction in dry matter content (Oladosu et al., 2016). In the current study, direct ensiling of untreated ryegrass resulted in high pH and low lactic acid concentration after fermentation and produced more butyric acid, which is consistent with the report of Li et al. (2019). In the present study, the different metabolism processes of different microbial communities at different harvests resulted in differences in pH values and organic acid concentrations.

Ensiling without the use of an inoculant or starter is a spontaneous fermentative process in which fermentation mainly depends on the composition of epiphytic microorganisms and the natural occurrence of epiphytic LAB (Guo et al., 2018). Many studies have shown that by using the PacBio SMRT sequencing, microorganism composition and its dynamics in the ensiling process can be precisely revealed at the species level because it can generate long sequence reads, which provides valuable biological information regarding the complete bacterial community in the ensiling system (Bai et al., 2021; Du et al., 2022a,b). The bacteria in all samples of fresh and silage ryegrass in this study were sequenced by SMRT sequencing technology to accurately assess the microbial community and diversity (Table 3). The coverage values were greater than 0.99 in all samples, which indicated that the sequencing depth was sufficient to detect the maximum bacterial communities. The Sobs diversity index in ryegrass silage was lower than that of fresh ryegrass, which indicated that the bacterial diversity of ryegrass decreased after ensiling. Alpha-diversity reflects the microbial abundance and species diversity of a single sample. Shannon’s index is used to measure species diversity, and a larger Shannon index indicated higher species diversity in the sample. Ace and Chao 1 indices are used to measure species richness, and lower Ace and Chao 1 index values indicated lower species richness of the sample (Zhang et al., 2020). Moreover, the Shannon index, Ace index, and Chao 1 index showed trends similar to the trend of the Sobs. This indicated that the bacterial diversity and abundance of ryegrass decreased after ensiling. Aerobic and facultative anaerobic fermentative bacteria consume oxygen and create an anaerobic environment. In general, during anaerobic fermentation, the metabolic activity of many undesirable microorganisms in raw materials is suppressed and gradually replaced by the LAB that dominate the fermentation process (Ni et al., 2017). This is similar to the results of Yan et al. (2019) who studied high-moisture Italian ryegrass silages. This may be due to the inability of some microorganisms to survive in anaerobic and acidic silage environments. Our findings are consistent with those of Ren et al. (2020), who discovered that the Chao 1 and Ace indices of sugarcane top silages were lowest at the end of fermentation, implying that the diversity of the microbial community may have declined as LAB became dominant in the silage. The Sobs, Shannon, Ace, and Chao 1 indices were lowest for the second harvest silage, which indicated that there may have been a small number of acidifying bacterial species in the second harvest silage (Zhang et al., 2020). The variation in the microbial community was further elucidated by analyzing the β-diversity of the bacterial population. In the PCoA plot, the variance in bacterial diversity was readily visible. Fresh forage and ensiled samples, as well as silages from different harvests, were effectively separated. This meant that the bacterial populations had altered during the ensiling process. The change in the bacterial population might explain differences in fermentation quality (Ni et al., 2017). In addition, the changes in metabolism before and after ensiling, and after ensiling different harvests were clearly demonstrated in the PCA plots. Metabolic profiles of fresh forage samples were successfully separated, but the metabolic profiles of first and third harvest silages overlapped, indicating the existence of the same metabolic pathway for both treatments.

The microbial community structure and species diversity are important factors affecting silage fermentation (Du et al., 2022a). As shown in Figure 2A, the Gram-negative bacteria *Pantoea* and unclassified bacteria were the main genera in the fresh materials, and *Pantoea vagans* were the main species. *Pantoea* is a genus of the *Erwiniaceae* family that was recently separated from the genus *Enterobacter*. *Pantoea* has a yellow pigment, ferments lactose, and forms mucoid colonies (Walterson and Stavrinides, 2015), and some *Pantoea* species are pathogenic to vegetables.

After fermentation, the main genera in silage were *Lactiplantibacillus*, *Enterococcus*, and a small amount of *Levilactobacillus*. During the fermentation process, *Lactobacillus*, *Lactococcus*, and *Enterococcus* species are desirable functional bacteria, and they have been routinely employed to improve silage quality (Chen et al., 2013; Yang et al., 2016). In addition, *Enterococcus* has been observed in high moisture environments, and it is generally held that in the fermentation process, *Enterococci*, *Lactococci*, *Pediococci*, and *Leuconostocs* commence silage fermentation, after which these bacteria are gradually replaced by more acid-tolerant *Lactobacilli* (e.g., *Lactiplantibacillus plantarum*) (Parvin et al., 2010). *Lactobacillus* was the most predominant microbe in all silage samples at any time point during the fermentation, according to Guan et al. (2018). At the same time, *Levilactobacillus* is hetero-fermentative lactate-producing bacteria found in various forage crops and silages. Their greater abundance is linked to increased lactic acid production, lower pH, and improved silage quality. This explains why in this study, the highest abundance of *Lactiplantibacillus plantarum* and *Levilactobacillus brevis* were found in the BK group, followed by the AK group, while the lowest abundance of *Lactiplantibacillus plantarum* and *Levilactobacillus brevis* was found in the CK group, noting that the order of treatments from low to high pH was BK, AK, and CK groups. *Lactiplantibacillus plantarum* plays an important role in inhibiting the growth of *Clostridium*, reducing ammonium nitrogen content, and improving fermentation quality (Du et al., 2022a). *Lactobacillus* was the most prevalent bacteria, accounting for 80–90% of the overall population in mixed silage of feed soybean and sorghum, according to Ni et al. (2018). Poor fermentation quality of alfalfa silage has been linked to a low relative abundance of *Lactobacillus* (11.7%) when unwanted bacteria predominate during ensiling (He et al., 2020). In general, desirable LAB, including *Lactiplantibacillus plantarum* and *Levilactobacillus brevis*, play a very important role in increasing lactic acid production and reducing pH value. Wang S. R. et al. (2020)) found that *Enterococcus* was frequently employed to improve fermentation characteristics. In the early stage of fermentation, *Enterococcus* might produce LA quickly and provide an acidic anaerobic environment to encourage LAB development, but *Enterococcus* is less resistant to acids, and when the acidic environment is too high, the growth of *Enterococcus* is inhibited (Cai et al., 1998; Nami et al., 2019). Similarly, in this study, compared with the BK group, the relative abundance of *Enterococcus* in CK and AK silages was higher, so the lactic acid content of CK and AK was lower than that of BK. Since *Enterococcus* are not acid-resistant genera and the pH value of BK was lower, there were few *Enterococcus* species in BK, and these were mostly *Lactiplantibacillus plantarum*, which were found to be more acid tolerant (Du et al., 2022b). In addition, more butyric acid and NH_3_-N accumulation in BK may have been due to higher protein content in the second ryegrass crop resulting in a higher protein degradation rate.

Proteobacteria was the dominant phylum in fresh ryegrass, which differs from that reported by Wang S. et al. (2020). This variation might be caused by the material’s chemical composition, as well as climate features (Guan et al., 2018). After ensiling, the relative abundance of Proteobacteria was obviously decreased, while the relative abundance of Firmicutes was obviously increased; at the same time, Proteobacteria, which is the most dominant phylum on fresh ryegrass, was quickly substituted by Firmicutes. Wang et al. (2022b) have shown similar results in Sudangrass silage. Firmicutes microorganisms are crucial acid-hydrolytic microbes under anaerobic circumstances containing anaerobic rumen and reactors, and cellulases, lipases, proteases, and other extracellular enzymes can be secreted by them (Wang S. et al., 2020; Wang et al., 2022c). Firmicutes quickly displaced the Proteobacteria phylum in the AK, BK, and CK groups after ensiling began. This was because the lower pH and anaerobic environment during ensiling favored the growth of Firmicutes species, which also explains the shift in the bacterial population from Proteobacteria to Firmicutes throughout the fermentation process in this study (Keshri et al., 2018). It is obvious that the BK group had the lowest pH value and the highest percentage of Firmicutes phylum, which supports this observation.

To assess the fermentation quality, many volatile organic acids can be analyzed in the silage, including lactic acid, acetic acid, propionic acid, and butyric acid (Weinberga and Muckb, 1996). However, more complex compounds can be produced by diverse microbial populations in natural or LAB-inoculated silages. Clearly, the restricted detection of numerous organic acids in silage cannot fully represent changes in their metabolites. The metabolomics technique can more accurately represent the composition of metabolites in the environment, and it has also been used for silage assessment (Guo et al., 2018; Xu et al., 2019). The current study is the first to reveal the metabolome of ryegrass silage using LC-MS metabolomics, which can detect the large molecular weight metabolites, and some metabolites of alfalfa silage have also been examined using this technique (Wang B. et al., 2020). In the present study, we used LEfSe to identify specific communities in the samples and found a significant enrichment of *Enterococcus hermanniensis* in AK, *Lactiplantibacillus plantarum* in BK, and Enterococcaceae in CK. Different harvests of ryegrass had different bacterial species, so different bacterial metabolic pathways lead to different metabolites produced during ensiling. After 60 days of ensiling, the most annotated metabolites were peptides, and eight amino acids were dominant in the composition of the identified peptides. The next most frequently annotated were carbohydrates, hormones, and transmitter metabolites. This suggests that the most important metabolic pathway of microorganisms during fermentation is amino acid metabolism, followed by carbohydrates and hormones and transmitter metabolisms. The raw material of the first harvest had a higher CP content (172.50 g/kg), and microorganisms in silage are prone to proteolysis and increased amino acid metabolism, suggesting that the amino acid metabolic pathways observed in silage may reflect the metabolic dynamics of the dominant microbial population in silage (Du et al., 2022a). Therefore, amino acid metabolism and carbohydrate metabolism are the main microbial metabolic pathways affecting the flavor and quality of silage due to protein hydrolysis and sugar degradation during fermentation (Du et al., 2021). Du et al. (2022a) reported that pyruvate metabolism, glycolysis, and butyrate metabolism dominated the carbohydrate metabolism pathways, which not only improved the fermentation quality of the silage but also the taste and palatability in animals. Moreover, our study showed that metabolic pathways, such as arginine biosynthesis; alanine, aspartate, and glutamate metabolism; glyoxylate and dicarboxylate metabolism; and purine metabolism were significantly affected by different harvests. Arginine, alanine, aspartic acid, and glutamic acid are all amino acids, which is consistent with the results of the highest number of amino acids among the compounds annotated by KEGG in Figure 5. Using this method not only greatly enriches the knowledge of metabolites in silage, but also helps to identify several metabolites that may be beneficial (Kamboh et al., 2015), and helps to mechanistically understand the effects of different treatments on metabolic pathways, thereby contributing to our understanding of the silage process.

This study also analyzed the association between the bacteria with high enrichment and taxonomic nomenclature in silage at different harvests and identified them as the top 30 most abundant metabolites. The findings of the present study in terms of the metabolite types and compositions of ryegrass silage were very different from the results of previous studies on whole crop corn silage and alfalfa silage (Guo et al., 2018; Xu et al., 2019). This might be due to the presence of different microbial communities in different forage species that produce different fermentation processes. Guan et al. (2020) found that the metabolites herniarin, tetrahydrocurcumin, glycerol, and dopamine were positively associated with *Lactobacillus*, and reported that the metabolites linked with the decrease of *Weissella* and the growth of *Acetobacter* were primarily odor-related acids and amines. Guo et al. (2018) reported that samples treated with inoculants resulted in an up-accumulation of several free amino acids. Some metabolites with antimicrobial activity were detected, such as 4-hydroxycinnamic acid, 3,4-dihydroxycinnamic acid, and catechol, as reported by Xu et al. (2019). In this study, the most annotated metabolites associated with the metabolic pathways relating to the two main genera *Lactiplantibacillus* and *Enterococcus* were amino acids, peptides, and analogs, which include valyl-isoleucine, D-pipecolic acid, isoleucyl-aspartate, L-phenylalanyl-L-proline, L-glutamic acid, and glutamylvaline. Amino acids play a crucial role in the life activities of living organisms, participating in the regulation of substance metabolism and information transfer in the living body, and they are the basic units of protein and an important indicator of the nutritional value of forage grasses. Peptides are intermediate products of protein hydrolysis. In this study, the valyl-isoleucine and glutamylvaline content increased with the increasing abundance of *Lactiplantibacillus plantarum*. The D-pipecolic acid and L-glutamic acid content increased with the increasing abundance of *Levilactobacillus brevis.* The L-phenylalanyl-L-proline content increased with the increasing abundance of *Enterococcus hermanniensis*. However, the isoleucyl-aspartate content decreased with the increasing abundance of *Enterococcus hermanniensis* and *Enterococcus faecalis*. The L-phenylalanyl-L-proline content decreased with the increasing abundance of *Levilactobacillus brevis*. The L-glutamic acid content decreased with the increasing abundance of *Enterococcus hermanniensis*. The glutamylvaline content decreased with the increasing abundance of *Enterococcus faecalis*. By understanding the relationship between these metabolites and bacteria, it may be possible to regulate the degradation of proteins during ensiling and reduce protein loss. Furthermore, some metabolites with carbohydrates and carbohydrate conjugates were detected, such as arabinonic acid, arabinofuranose, and 3,4,5-trihydroxy-6-(2-methoxybenzoyloxy) oxane-2-carboxylic acid. 3,4,5-Trihydroxy-6-(2-methoxybenzoyloxy) oxane-2-carboxylic acid content increased with the increasing abundance of *Enterococcus hermanniensis*. However, the arabinonic acid content decreased with the increasing abundance of *Enterococcus faecalis*. Arabinofuranose content decreased with the increasing abundance of *Enterococcus hermanniensis*. 3,4,5-Trihydroxy-6-(2-methoxybenzoyloxy) oxane-2-carboxylic acid content decreased with increasing abundance of *Levilactobacillus brevis*. Understanding the relationship between metabolites and fermentation bacteria may improve the quality of silage by regulating the fermentation process. In addition, some metabolites with alcohols and polyols were detected in silage samples, such as chlorogenic acid, 1,3-dicaffeoylquinic acid, and shikimic acid. Chlorogenic acid content increased with the increasing abundance of *Enterococcus faecalis*, and the shikimic acid content increased with the increasing abundance of *Enterococcus hermanniensis*. However, the chlorogenic acid and 1,3-dicaffeoylquinic acid content decreased with the increasing abundance of *Enterococcus faecalis*. Shikimic acid content decreased with the increasing abundance of *Levilactobacillus brevis*. *Lactiplantibacillus plantarum* and *Levilactobacillus brevis* harbored a high abundance of genes encoding for ethanol dehydrogenase, which is involved in the production of aroma compounds (Tlais et al., 2022a). Ethanol content is one of the main bases for assessing silage fermentation (Wang et al., 2022c), and the perception of a sour taste can be affected by the presence of polyols, in addition to organic acids (Tlais et al., 2022b). Understanding the relationship between metabolites with alcohols and polyols and fermentation bacteria can provide better insight into the fermentation mechanism of silage.

## Conclusion

The present study suggested that different harvests were associated with differences in the bacterial community and metabolomic profile of ryegrass silage, thus affecting fermentation quality. Silage of the second harvest had a lower pH and higher abundance of *Lactiplantibacillus* after ensiling. The dominant bacteria in naturally fermented ryegrass after silage were *Lactiplantibacillus* and *Enterococcus*, and the most annotated metabolites were peptides, and eight amino acids were dominant in the composition of the identified peptides. Different harvests mainly affected the metabolism of arginine biosynthesis and alanine, aspartate, and glutamate metabolism pathway. Valyl-isoleucine and glutamylvaline content increased with increasing abundance of *Lactiplantibacillus plantarum.* The content of D-pipecolic acid and L-glutamic acid increased with increasing abundance of *Levilactobacillus brevis*, and the L-phenylalanyl-L-proline, 3,4,5-trihydroxy-6-(2-methoxybenzoyloxy) oxane-2-carboxylic acid, and shikimic acid content decreased with increasing abundance of *Levilactobacillus brevis*. Therefore, the use of a combined analysis of silage microorganisms and metabolome provides a deeper understanding of the fermentation mechanism of silage. This provides new information for regulating ryegrass silage fermentation processes, which can contribute to improving silage quality and utilization.

## Data availability statement

The datasets generated for this study can be found in the NCBI under accession number SRP372136.

## Author contributions

ZF contributed to methodology, visualization, validation, and data curation and wrote the original draft. LS and QL interpreted the data and edited the language. YJ, ZW, TL, and XR contributed to conceptualization, acquisition, reviewing, and editing. MH and JH contributed to software. GG contributed to conceptualization and funding acquisition. All authors have read and agreed to the published version of the manuscript.

## Footnotes

1
http://www.genome.jp/kegg/

2
https://docs.scipy.org/doc/scipy/

3
www.majorbio.com

## References

[B1] Amir A. Zeisel A. Zuk O. Elgart M. Stern S. Shamir O. (2013). High-resolution microbial community reconstruction by integrating short reads from multiple 16S rRNA regions. *Nucleic Acids Res.* 41:205. 10.1093/nar/gkt1070 24214960 PMC3905898

[B2] Bai J. Ding Z. Ke W. Xu D. Wang M. Huang W. (2021). Different lactic acid bacteria and their combinations regulated the fermentation process of ensiled alfalfa: Ensiling characteristics, dynamics of bacterial community and their functional shifts. *Microbiol. Biotechnol.* 14 1171–1182. 10.1111/1751-7915.13785 33666350 PMC8085944

[B3] Broderick G. A. Kang J. H. (1980). Automated simultaneous determination of ammonia and total amino acids in ruminal fluid and in vitro media. *J. Dairy Sci.* 63 64–75. 10.3168/jds.S0022-0302(80)82888-8 7372898

[B4] Cai Y. (2004). *Analysis Method for Silage Japanese Society of Grassland Science Field and Laboratory Methods for Grassland Science.* Tokyo: Tosho Printing Co., Ltd, 279–282.

[B5] Cai Y. M. Benno Y. Ogawa M. Kumai S. (1999). Effect of applying lactic acid bacteria isolated from forage crops on fermentation characteristics and aerobic deterioration of silage. *J. Dairy Sci.* 82 520–526. 10.3168/jds.S0022-0302(99)75263-X 10194670

[B6] Cai Y. Benno Y. Ogawa M. Ohmomo S. Kumai S. Nakase T. (1998). Influence of *Lactobacillus* spp. from an inoculant and of *Weissella* and *Leuconostoc* spp. from forage crops on silage fermentation. *Appl. Environ. Microbiol.* 64 82–87. 10.1128/AEM.64.8.2982-2987.1998 9687461 PMC106803

[B7] Chen L. Guo G. Yu C. Zhang J. Shimojo M. Shao T. (2015). The effects of replacement of whole-plant corn with oat and common vetch on the fermentation quality, chemical composition and aerobic stability of total mixed ration silage in Tibet. *Anim. Sci. J.* 86 69–76. 10.1111/asj.12245 25091371

[B8] Chen M. M. Liu Q. H. Xin G. R. Zhang J. G. (2013). Characteristics of lactic acid bacteria isolates and their inoculating effects on the silage fermentation at high temperature. *Lett. Appl. Microbiol.* 56 71–78. 10.1111/lam.12018 23106758

[B9] Cheng Q. M. Li P. Xiao B. Yang F. Li D. Ge G. T. (2021). Effects of LAB inoculant and cellulase on the fermentation quality and chemical composition of forage soybean silage prepared with corn stover. *Grassl. Sci.* 67 83–90. 10.1111/grs.12289

[B10] Ding Z. T. Bai J. Xu D. Li F. Zhang Y. Guo X. (2020). Microbial community dynamics and natural fermentation profiles of ensiled alpine grass *Elymus nutans* prepared from different regions of the qinghai-tibetan plateau. *Front. Microbiol.* 11:855. 10.3389/fmicb.2020.00855 32477296 PMC7235320

[B11] Dong L. F. Zhang H. S. Gao Y. H. Diao Q. Y. (2020). Dynamic profiles of fermentation characteristics and bacterial community composition of *Broussonetia papyrifera* ensiled with perennial ryegrass. *Bioresour. Technol.* 310:123396. 10.1016/j.biortech.2020.123396 32388351

[B12] Du Z. Sun L. Chen C. Lin J. Yang F. Cai Y. (2021). Exploring microbial community structure and metabolic gene clusters during silage fermentation of paper mulberry, a high-protein woody plant. *Anim. Feed Sci. Technol*. 275:114766. 10.1016/j.anifeedsci.2020.114766

[B13] Du Z. Sun L. Lin Y. Chen C. Yang F. Cai Y. (2022a). Use of Napier grass and rice straw hay as exogenous additive improves microbial community and fermentation quality of paper mulberry silage. *Anim. Feed Sci. Tech.* 285:115219. 10.1016/j.anifeedsci.2022.115219

[B14] Du Z. Yamasaki S. Oya T. Nguluve D. Euridse D. Tinga B. (2022b). Microbial co-occurrence network and fermentation information of natural woody-plant silage prepared with grass and crop by-product in Southern Africa. *Front. Microbiol.* 13:756209. 10.3389/fmicb.2022.756209 35369476 PMC8964296

[B15] Guan H. Shuai Y. Ren Q. Yan Y. Wang X. Li D. (2020). The microbiome and metabolome of Napier grass silages prepared with screened lactic acid bacteria during ensiling and aerobic exposure. *Anim. Feed Sci. Tech.* 269:114673. 10.1016/j.anifeedsci.2020.114673

[B16] Guan H. Yan Y. Li X. Li X. M. Shuai Y. Feng G. (2018). Microbial communities and natural fermentation of corn silages prepared with farm bunker-silo in Southwest China. *Bioresour. Technol.* 265 282–290. 10.1016/j.biortech.2018.06.018 29908496

[B17] Guo X. Ke X. Ding W. Ding L. Xu D. Wang W. (2018). Profiling of metabolome and bacterial community dynamics in ensiled *Medicago sativa* inoculated without or with *Lactobacillus plantarum* or *Lactobacillus buchneri*. *Sci. Rep.* 8:357. 10.1038/s41598-017-18348-0 29321642 PMC5762819

[B18] He L. Lv H. Xing Y. Wang C. You X. Chen X. (2020). The nutrients in Moringa oleifera leaf contribute to the improvement of stylo and alfalfa silage: Fermentation, nutrition and bacterial community. *Bioresour. Technol.* 301:122733. 10.1016/j.biortech.2020.122733 31935644

[B19] Hou Q. C. Xu H. Zheng Y. Xi X. Kwok L. Y. Sun Z. (2015). Evaluation of bacterial contamination in raw milk, ultra-high temperature milk and infant formula using single molecule, real-time sequencing technology. *J. Dairy Sci.* 98 8464–8472. 10.3168/jds.2015-9886 26476945

[B20] Kamboh A. Arain M. Mughal M. Zaman A. Arain Z. Soomro A. (2015). Flavonoids: Health promoting phytochemicals for animal production-a review. *J. Anim. Health Prod.* 3 6–13. 10.14737/journal.jahp/2015/3.1.6.13

[B21] Keles G. Kurtoglu V. Demirci U. Ates S. Canatan T. Kan M. (2014). Conservation characteristics of triticale-Hungarian vetch silage ensiled with homo-fermentative or hetero-fermentative lactic acid bacteria in jars. *Anim. Nutr. Feed Technol.* 14 69–79.

[B22] Keshri J. Chen Y. Pinto R. Kroupitski Y. Weinberg Z. G. Sela Saldinger S. (2018). Microbiome dynamics during ensiling of corn with and without *Lactobacillus plantarum* inoculant. *Appl. Microbiol. Biotechnol.* 102 4025–4037. 10.1007/s00253-018-8903-y 29536147

[B23] Kung L. M. Shaver R. D. Grant R. J. Schmidt R. J. (2018). Silage review: Interpretation of chemical, microbial, and organoleptic components of silages. *J. Dairy Sci.* 101 4020–4033. 10.3168/jds.2017-13909 29685275

[B24] Li P. Zhang Y. Gou W. Cheng Q. Bai S. Cai Y. (2019). Silage fermentation and bacterial community of bur clover, annual ryegrass and their mixtures prepared with microbial inoculant and chemical additive. *Anim. Feed Sci. Tech.* 247 285–293. 10.1016/j.anifeedsci.2018.11.009

[B25] Li Y. Du S. Sun L. Cheng Q. Hao J. Lu Q. (2022). Effects of lactic acid bacteria and molasses additives on dynamic fermentation quality and microbial community of native grass silage. *Front. Microbiol.* 13:830121. 10.3389/fmicb.2022.830121 35401455 PMC8989346

[B26] Lv R. L. Elsabagh M. Obitsu T. Sugino T. Kurokawa Y. (2021). Effect of phytol in forage on phytanic acid content in cow’s milk. *Anim. Biosci.* 34 1616–1622. 10.5713/ab.21.0086 34237930 PMC8495353

[B27] Magoc T. Salzberg S. L. (2011). FLASH: Fast length adjustment of short reads to improve genome assemblies. *Bioinformatics* 27 2957–2963. 10.1093/bioinformatics/btr507 21903629 PMC3198573

[B28] Mcdonald P. Henderson A. R. Heron S. (1991). *The Biochemistry of Silage*, 2nd Edn. Marlow: Chalcombe Publications.

[B29] Nami Y. Bakhshayesh R. V. Jalaly H. M. Lotfi H. Eslami S. Hejazi M. A. (2019). Probiotic properties of *Enterococcus* isolated from artisanal dairy products. *Front Microbiol.* 10:300. 10.3389/fmicb.2019.00300 30863379 PMC6400110

[B30] Ni K. K. Wang F. Zhu B. Yang J. Zhou G. Pan Y. (2017). Effects of lactic acid bacteria and molasses additives on the microbial community and fermentation quality of soybean silage. *Bioresour. Technol.* 238 706–715. 10.1016/j.biortech.2017.04.055 28501002

[B31] Ni K. K. Zhao J. Zhu B. Su R. Pan Y. Ma J. (2018). Assessing the fermentation quality and microbial community of the mixed silage of forage soybean with crop corn or sorghum. *Bioresour. Technol.* 265 563–567. 10.1016/j.biortech.2018.05.097 29861298

[B32] Oladosu Y. Rafii M. Y. Abdullah N. Magaji U. Hussin G. Ramli A. (2016). Fermentation quality and additives: A case of rice straw silage. *Biomed. Res. Int.* 2016:7985167. 10.1155/2016/7985167 27429981 PMC4939334

[B33] Parvin S. Wang C. Li Y. Nishino N. (2010). Effects of inoculation with lactic acid bacteria on the bacterial communities of Italian ryegrass, whole crop maize, guinea grass and rhodes grass silages. *Anim. Feed Sci. Tech.* 160 160–166. 10.1016/j.anifeedsci.2010.07.010

[B34] Patrica C. (1997). *Official Method of Analysis of AOAC International*, 16th Edn. Washington, DC: AOAC International.

[B35] Ren H. Feng Y. Pei J. Li J. Wang Z. Fu S. (2020). Effects of *Lactobacillus plantarum* additive and temperature on the ensiling quality and microbial community dynamics of cauliflower leaf silages. *Bioresour. Technol.* 307:123238. 10.1016/j.biortech.2020.123238 32247271

[B36] Schloss P. D. Jenior M. L. Koumpouras C. C. Westcott S. L. Highlander S. K. (2016). Sequencing 16S rRNA gene fragments using the PacBio SMRT DNA sequencing system. *PeerJ* 4 1869–1885. 10.7717/peerj.1869 27069806 PMC4824876

[B37] Smit H. J. Tas B. M. Taweel H. Z. Tamminga S. Elgersma A. (2005). Effects of perennial ryegrass (Lolium perenne L.) cultivars on herbage production, nutritional quality and herbage intake of grazing dairy cows. *Grass Forage Sci.* 60 297–309. 10.1111/j.1365-2494.2005.00480.x

[B38] Stergiadis S. Allen M. Chen X. J. Wills D. Yan T. (2015). Prediction of nutrient digestibility and energy concentrations in fresh grass using nutrient composition. *J. Dairy Sci.* 98 3257–3273. 10.3168/jds.2014-8587 25747838

[B39] Sun L. Na N. Li X. Li Z. Wang C. Wu X. (2021). Impact of packing density on the bacterial community, fermentation, and in vitro digestibility of whole-crop barley silage. *Agric. Basel.* 11:672. 10.3390/agriculture11070672

[B40] Sun M. C. Li A. L. Huo G. C. Meng X. C. (2012). Progress on the metabolomics of lactic acid bacteria. *Microbiol. Tongbao* 39 1499–1505. 10.1371/journal.pone.0190040 29298316 PMC5752023

[B41] Tlais A. Junior W. Filannino P. Campanaro S. Gobbetti M. Cagno R. (2022a). How microbiome composition correlates with biochemical changes during sauerkraut fermentation: A focus on neglected bacterial players and functionalities. *Microbiol. Spectrum* e0016822. Online ahead of print. 10.1128/spectrum.00168-22 35699432 PMC9430578

[B42] Tlais A. Kanwal S. Filannino P. Albiac M. Gobbetti M. Cagno R. (2022b). Effect of sequential or ternary starters-assisted fermentation on the phenolic and glucosinolate profiles of sauerkraut in comparison with spontaneous fermentation. *Food Res. Int.* 156:111116. 10.1016/j.foodres.2022.111116 35650999

[B43] Van S. P. V. Robertson J. B. Lewis B. A. (1991). Methods for dietary fiber, neutral detergent fiber, and nonstarch polysaccharides in relation to animal nutrition. *J. Dairy Sci.* 74 83–97. 10.3168/jds.S0022-0302(91)78551-2 1660498

[B44] Walterson A. M. Stavrinides J. (2015). Pantoea: Insights into a highly versatile and diverse genus within the *Enterobacteriaceae*. *FEMS Microbiol. Rev.* 39 968–984. 10.1093/femsre/fuv027 26109597

[B45] Wang B. Gao R. Wu Z. Yu Z. (2020). Functional analysis of sugars in modulating bacterial communities and metabolomics profiles of medicago sativa silage. *Front. Microbiol.* 11:641. 10.3389/fmicb.2020.00641 32477276 PMC7232540

[B46] Wang Q. Garrity G. M. Tiedje J. M. Cole J. R. (2007). Naive Bayesian classifier for rapid assignment of rRNA sequences into the new bacterial taxonomy. *Appl. Environ. Microbiol.* 73 5261–5267. 10.1128/Aem.00062-07 17586664 PMC1950982

[B47] Wang S. R. Li J. Zhao J. Dong Z. Shao T. (2022b). An investigation of fermentative profile, microbial numbers, bacterial community diversity and their predicted metabolic characteristics in Sudangrass (*Sorghum sudanense* Stapf.) silages. *Anim. Biosci*. 35 1162–1173. 10.5713/ab.21.0326 34991212 PMC9262718

[B48] Wang S. R. Li J. Zhao J. Dong Z. Shao T. (2022c). Effect of storage time on the fermentation quality, bacterial community structure and metabolic profiles of napiergrass (*Pennisetum purpureum* Schum.) silage. *Arch. Microbiol.* 204:22. 10.1007/s00203-021-02658-z 34913097

[B49] Wang S. R. Li J. Zhao J. Dong Z. Dong D. Shao T. (2022a). Dynamics of the bacterial communities and predicted functional profiles in wilted alfalfa silage. *J. Appl. Microbiol.* 132 2613–2624. 10.1111/jam.15417 34923727

[B50] Wang S. R. Yuan X. J. Dong Z. H. Li J. F. Shao T. (2017). Effect of ensiling corn stover with legume herbages in different proportions on fermentation characteristics, nutritive quality and in vitro digestibility on the Tibetan Plateau. *Grassl. Sci.* 63 236–244. 10.1111/grs.12173

[B51] Wang S. R. Zhao J. Dong Z. Li J. Kaka N. A. Shao T. (2020). Sequencing and microbiota transplantation to determine the role of microbiota on the fermentation type of oat silage. *Bioresource Technol.* 309:123371. 10.1016/j.biortech.2020.123371 32305853

[B52] Wang S. Dong Z. Li J. Chen L. Shao T. (2018). Effects of storage temperature and combined microbial inoculants on fermentation end products and microbial populations of Italian ryegrass (*Lolium multiflorum* Lam.) silage. *J. Appl. Microbiol.* 125 1682–1691. 10.1111/jam.14083 30133082

[B53] Wang S. Sun Y. Zhao J. Dong Z. Li J. Nazar M. (2020). Assessment of inoculating various epiphytic microbiota on fermentative profile and microbial community dynamics in sterile Italian ryegrass. *J. Appl. Microbiol.* 129 509–520. 10.1111/jam.14636 32167651

[B54] Weinberga Z. G. Muckb R. E. (1996). New trends and opportunities in the development and use of inoculants for silage. *FEMS Microbiol. Rev.* 19 53–68. 10.1016/0168-6445(96)00025-3

[B55] Wright D. A. Gordon F. J. Steen R. W. J. Patterson D. C. (2000). Factors influencing the response in intake of silage and animal performance after wilting of grass before ensiling: A review. *Grass Forage Sci.* 55 1–3. 10.1046/j.1365-2494.2000.00198.x

[B56] Xu D. Ding W. Ke W. Li F. Zhang P. Guo X. (2019). Modulation of metabolome and bacterial community in whole crop corn silage by inoculating homofermentative *Lactobacillus plantarum* and heterofermentative *Lactobacillus buchneri*. *Front. Microbiol.* 9:3299. 10.3389/fmicb.2018.03299 30728817 PMC6352740

[B57] Yan Y. Li X. Guan H. Huang L. Ma X. Peng Y. (2019). Microbial community and fermentation characteristic of Italian ryegrass silage prepared with corn stover and lactic acid bacteria. *Bioresour. Technol.* 279 166–173. 10.1016/j.biortech.2019.01.107 30721817

[B58] Yang J. S. Tan H. S. Cai Y. M. (2016). Characteristics of lactic acid bacteria isolates and their effect on silage fermentation of fruit residues. *J. Dairy Sci.* 99 5325–5334. 10.3168/jds.2016-10952 27108171

[B59] Yang Z. Wang D. Li Y. Zhou X. Liu T. Shi C. (2022). Untargeted metabolomics analysis of the anti-diabetic effect of red ginseng extract in Type 2 diabetes mellitus rats based on UHPLC-MS/MS. *Biomed. Pharmacother.* 146:112495. 10.1016/j.biopha.2021.112495 34891123

[B60] Zhang G. Fang X. Feng G. Li Y. Zhang Y. (2020). Silage fermentation, bacterial community, and aerobic stability of total mixed ration containing wet corn gluten feed and corn stover prepared with different additives. *Animals* 10:1775. 10.3390/ani10101775 33019521 PMC7599836

[B61] Zhang Q. Wu B. Y. L. Nishino N. Wang X. G. Yu Z. (2016). Fermentation and microbial population dynamics during the ensiling of native grass and subsequent exposure to air. *Anim. Sci. J.* 87 389–397. 10.1111/asj.12427 26950516

[B62] Zhang W. Tan B. Ye G. Wang J. Dong X. Yang Q. (2019). Identification of potential biomarkers for soybean meal-induced enteritis in juvenile pearl gentian grouper, Epinephelus lanceolatus♂× Epinephelus fuscoguttatus♀. *Aquaculture* 512:734337. 10.1016/j.aquaculture.2019.734337

